# Supplementary figures and images for: Human FcRn expression and Type I Interferon signaling control Echovirus 11 pathogenesis in mice
Source: PLoS Pathog. 2021 Jan 29;17(1):e1009252. doi: 10.1371/journal.ppat.1009252 (PMC7875378; doi:10.1371/journal.ppat.1009252)

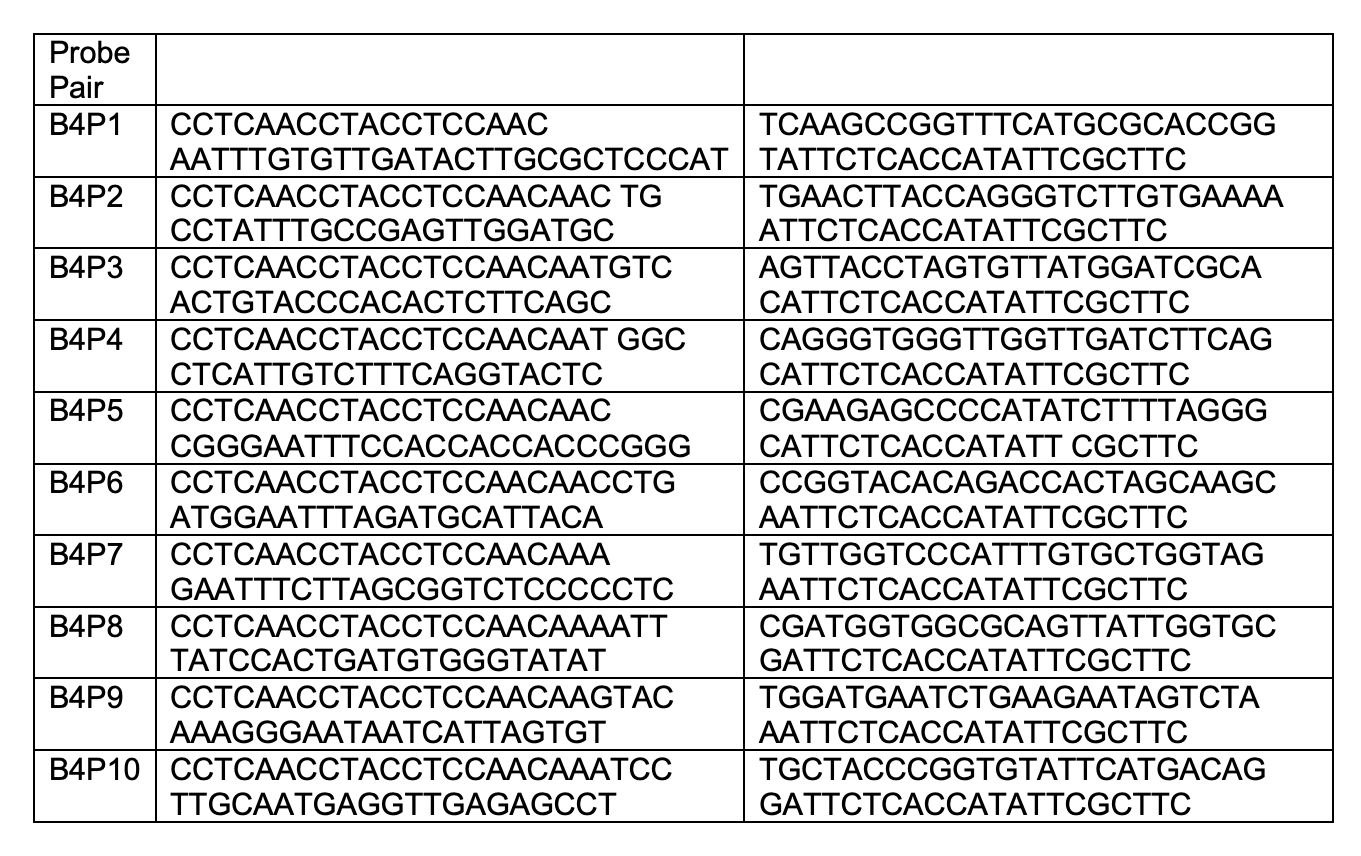

Supplement: S1 Table — (TIF) [file ppat.1009252.s001.tif]

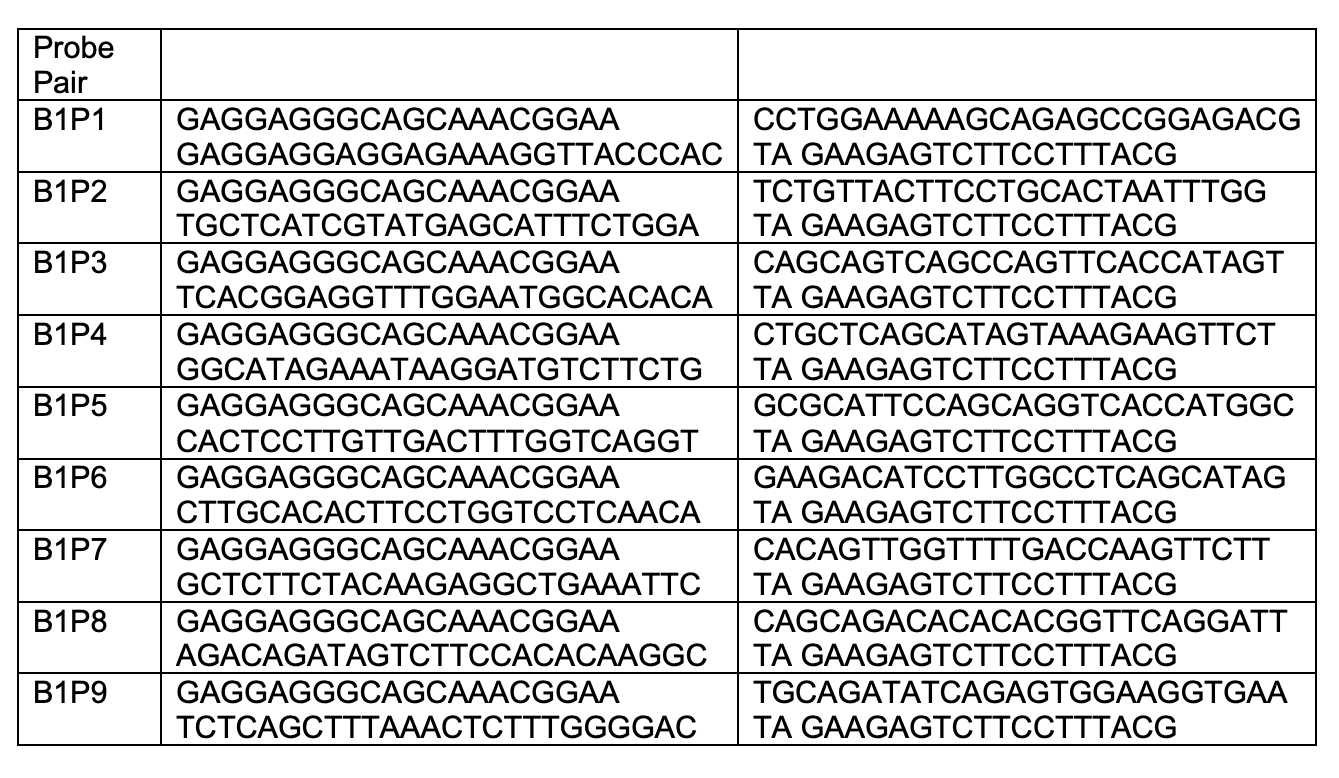

Supplement: S2 Table — (TIF) [file ppat.1009252.s002.tif]

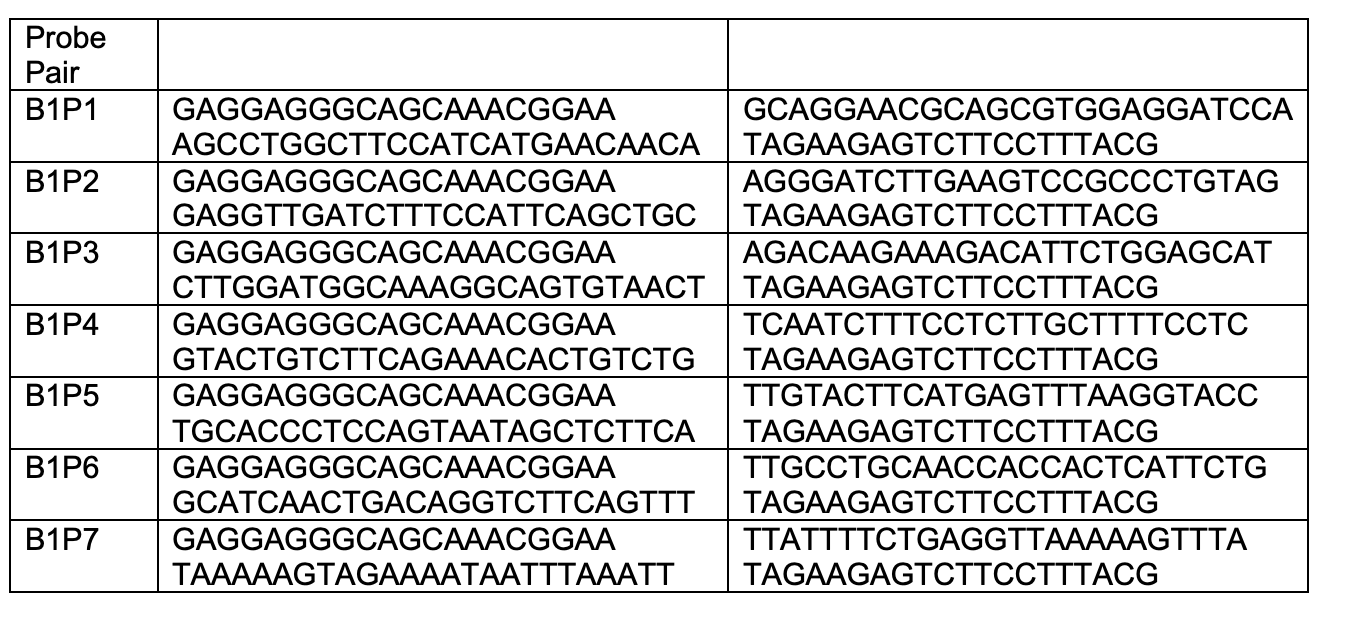

Supplement: S3 Table — (TIF) [file ppat.1009252.s003.tif]

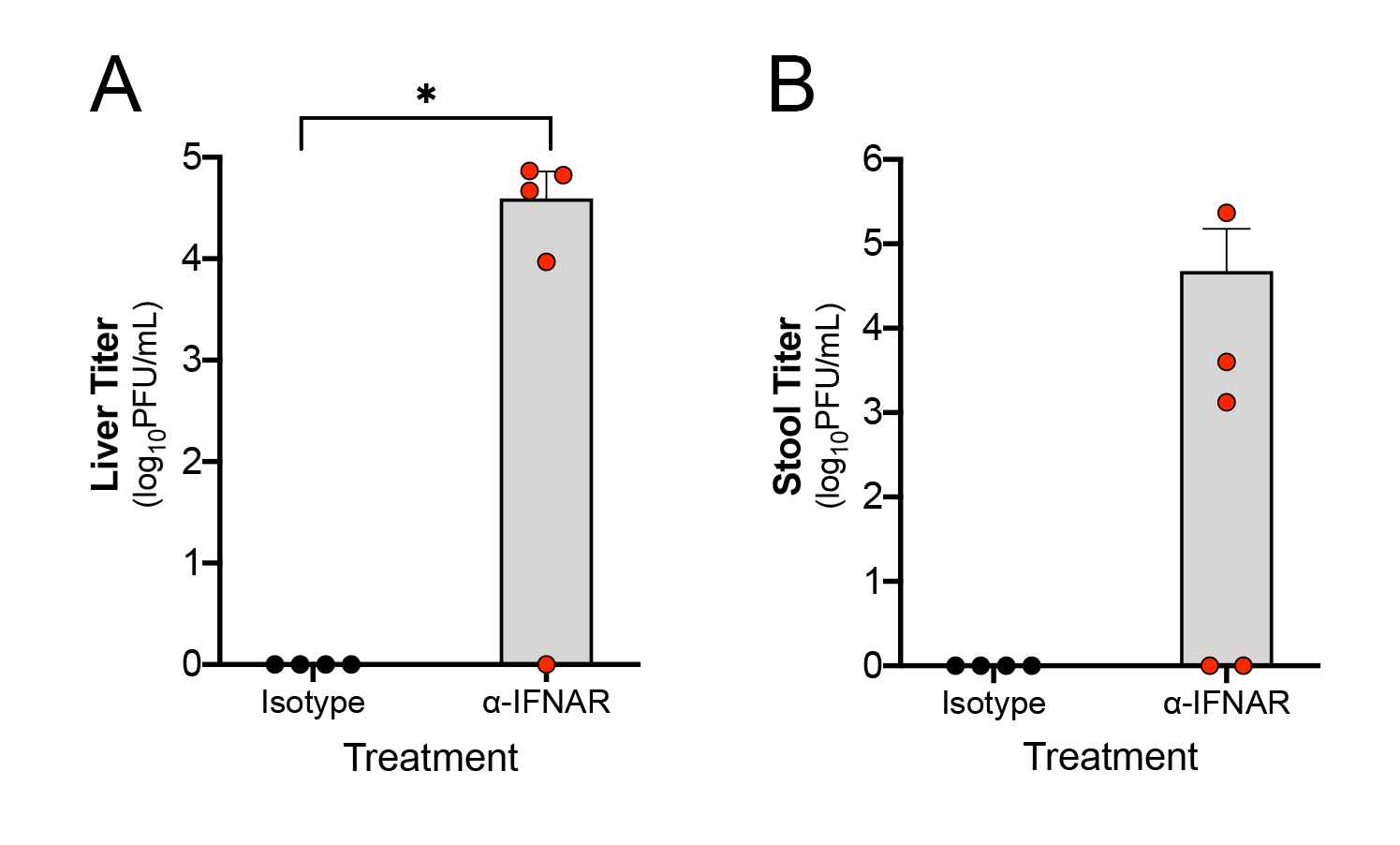

Supplement: S1 Fig — Viral titers in the liver (A) or stool (B). Data are shown with significance determined with a Mann-Whitney test (*p<0.05). (TIF) [file ppat.1009252.s004.tif]

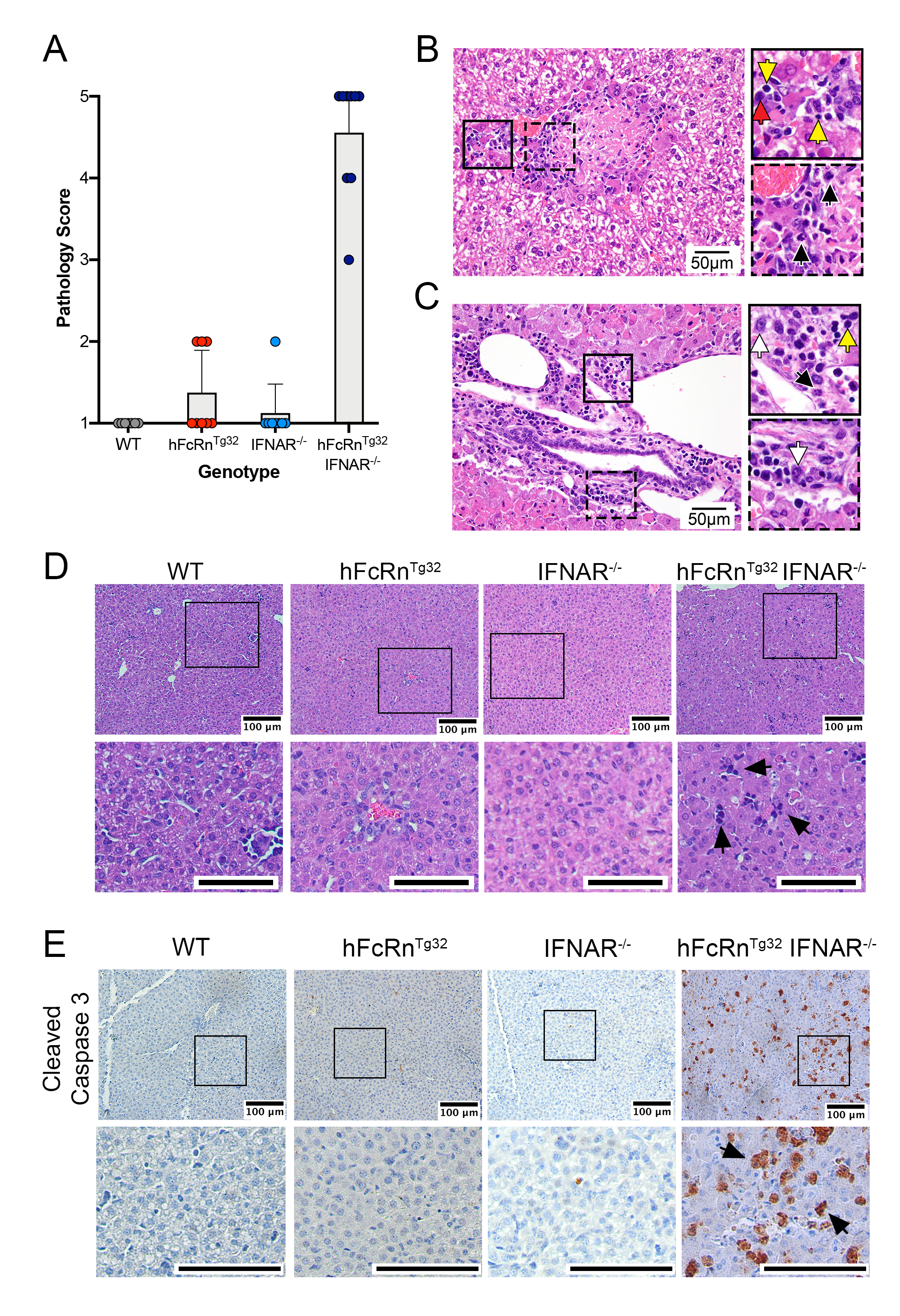

Supplement: S2 Fig — H&E sections were scored blinded to genotype based on severity of pathology using the following descriptors—1: retention of normal architecture and cord pattern of liver cells, 2: Immune infiltration, 3: spotty/random hepatocytolysis, 4: punctate aggregates of hepatocyte necrosis/death, and 5: confluent areas of hepatocyte necrosis and death. (B) Representative image of an hFcRnTg32-IFNAR-/- adult animal with areas of necrosis are often associated with neutrophils (black arrows) around the edge, along with lymphocytes (yellow arrows), and plasma cells (red arrows). (C) Representative image of an hFcRnTg32-IFNAR-/- adult animal around the portal areas with macrophages (white arrows), lymphocytes (yellow arrows), and neutrophils (black arrows). (D) H&E staining of the livers in suckling mice. C57Bl/6 (WT), hFcRnTg32, IFNAR-/-, and hFcRnTg32-IFNAR-/- suckling mice were IP inoculated with 104 E11 and sacrificed 72 hours post inoculation. Black arrows denote areas of immune infiltration. (E) Immunohistochemistry using an antibody recognizing the cleaved form of caspase 3 from the livers of a representative suckling mouse of each genotype as indicated. Black arrows denote positive staining. Scale bars (100μm) are shown at bottom right. (TIF) [file ppat.1009252.s005.tif]

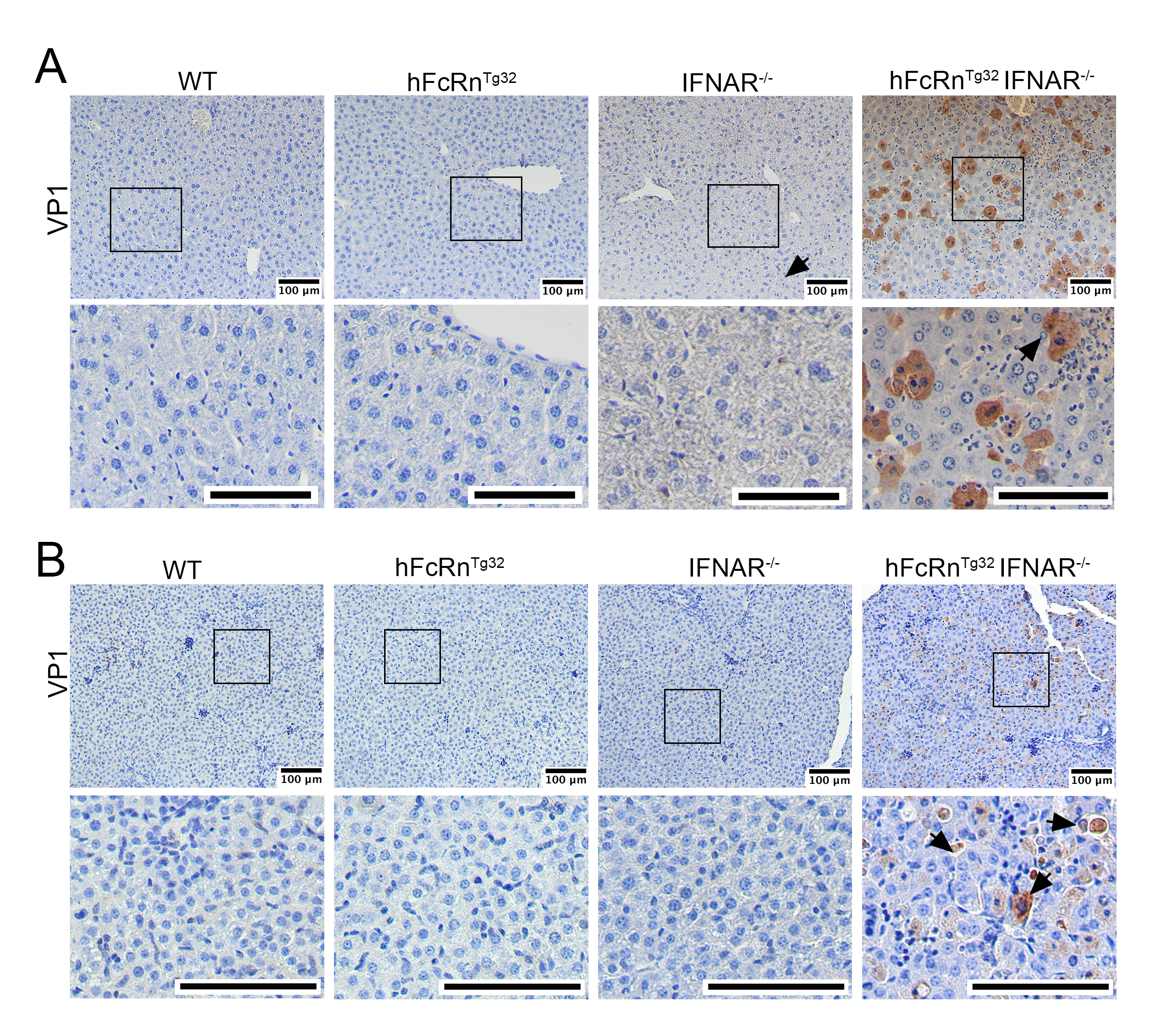

Supplement: S3 Fig — WT, hFcRnTg32, IFNAR-/-, and hFcRnTg32-IFNAR adult (A) or suckling mice (B) were inoculated with E11 by the IP route and sacrificed 72 hours post-inoculation. Shown are representative images from immunohistochemistry for E11 using an antibody recognizing the VP1 capsid protein from the livers of a representative animal of each genotype. (TIF) [file ppat.1009252.s006.tif]
